# Managing Longevity Risks: Consumer Attitudes Toward and Experiences With Financial Products

**DOI:** 10.1093/geroni/igaf122.1988

**Published:** 2025-12-31

**Authors:** Chaiwoo Lee, Kathryn Warren, Lisa D’Ambrosio, Sophia Ashebir, Joseph Coughlin

**Affiliations:** Massachusetts Institute of Technology, Cambridge, Massachusetts, United States; MIT AgeLab, Cambridge, Massachusetts, United States; Massachusetts Institute of Technology, Cambridge, Massachusetts, United States; Massachusetts Institute of Technology, Cambridge, Massachusetts, United States; Massachusetts Institute of Technology AgeLab, Cambridge, Massachusetts, United States

## Abstract

Financial products such as annuities, life insurance, and disability insurance are positioned to play a key role in future planning, including retirement, by providing financial security; hedging against different kinds of financial risks, including illness, injury, or death; and maintaining a steady income stream throughout older age. A national online survey of employed American adults ages 25 to 67, with medium to high income or investable assets, explored consumers’ sense of preparedness for their financial futures and lives in older age. Items included people’s overall financial well-being; attitudes toward and usage of various financial products; attitudes toward different longevity risks; and expectations for retirement. Data on demographic characteristics, recent and anticipated life events, and behavioral characteristics such as risk attitudes and optimism were also gathered to allow for multidimensional comparisons. Preliminary results indicate that while concerns around costs related to health conditions and disability, income stability, and spending flexibility are prevalent among consumers, adoption rates for annuities and disability insurance remain low. Across the three products covered in the survey, usage was significantly correlated with knowledge of and familiarity with the product. Many people were learning about these products from informal sources such as family and friends, and through internet searches, rather than from financial institutions or employers. This presentation will provide a comprehensive look into how different financial products and concerns to manage different longevity risks fit into people’s future planning, and how people’s preferences and experiences differ by their family situation or by individual characteristics.

